# LEUKOENCEPHALOPATHY WITH EVANESCENT WHITE MATTER: A CASE REPORT

**DOI:** 10.1590/1984-0462/;2018;36;4;00001

**Published:** 2018

**Authors:** Renata Porciuncula, Patricia Kelly Wilmsen Dalla Santa Spada, Karen Olivia Bazzo Goulart

**Affiliations:** a FSG Centro Universitário da Serra Gaúcha, Caxias do Sul, RS, Brasil.

**Keywords:** Leukoencephalopathy with vanishing white matter, Genetics, Central nervous system, Child, Leucoencefalopatia com substância branca evanescente, Genética, Sistema nervoso central, Criança

## Abstract

**Objective:**

To describe the case of a child diagnosed with leukoencephalopathy with vanishing white matter (LVWM), a rare genetic disease with autosomal recessive inheritance pattern.

**Case description:**

A 5-month-old male child started to refuse breast-feeding, showing somnolence and signs of dehydration,with dry mouth, increasing body temperature and adipsy. As days went by, the symptoms got worse. The infant was very sleepy and was transferred to the intensive care unit, where he stayed for one week. At this time, a signal alteration with hyper attenuated T2 predominance was identified in the magnetic resonance imaging, compromising the white matter, which had diffuse and symmetrical aspect. At this time, the infant started to present seizures. When the infant was 11 months old, he was diagnosed with tonsillitis and presented recurrent fever peaks and extreme sleepiness. After hospital admission, the infant progressed to a comatose state and died. The diagnosis of LVWM was confirmed in examinations performed after death. As a late diagnosis, a genetic disease was identified with a mutation in one of the five genes responsible for the codification of complex eukaryotic translation initiation factor 2B (eIF2B), involved with the control of the protein translation and which is described as pathogenic in individuals with LVWM.

**Comments:**

LVWM is a hereditary brain disease that occurs primarily in children. The disease is chronic and progressive, with additional episodes of rapid deterioration, as shown in the present case report.

## RESUMO

**Objetivo:**

Descrever uma criança diagnosticada com leucoencefalopatia com substância branca evanescente (LSBE), uma doença genética rara que possui padrão de herança autossômico recessivo.

**Descrição do caso:**

Criança do sexo masculino, com 5 meses de idade, que mostrava recusa da amamentação e sonolência, começou a apresentar quadro de desidratação, com boca seca, aumento da temperatura corporal e adipsia. Com o passar dos dias, os sintomas agravaram-se. O lactente apresentou-se muito sonolento e foi transferido para a unidade de tratamento intensivo (UTI), onde permaneceu por uma semana. Nesse período, foi identificada, na ressonância magnética de crânio, uma alteração de sinal com predomínio hiperatenuado T2, comprometendo particularmente a substância branca, de aspecto difuso e simétrico. O lactente apresentou crises convulsivas desde então. Aos 11 meses foi diagnosticado com tonsilite, demonstrando quadros recorrentes de picos febris e sonolência excessiva. Na evolução do quadro, o lactente entrou em estado comatoso progredindo a óbito. O diagnóstico de LSBE foi confirmado em exames realizados após o óbito, e tardiamente foi identificada uma doença genética decorrente de mutações em um dos cinco genes que são responsáveis pela codificação do complexo fator de iniciação da tradução de eucariontes 2B (eIF2B), envolvido com o controle da tradução de proteínas, sendo descrita como patogênica em indivíduos com LSBE.

**Comentários:**

A LSBE é uma doença cerebral hereditária com início na infância. A doença apresenta-se de maneira crônica e progressiva, com episódios adicionais de rápida deterioração, como evidenciado no presente relato de caso.

## INTRODUCTION

Leukoencephalopathy with vanishing white matter (LVWM) is a central hereditary brain disease which affects mainly children. The disease presents chronically and progressively, with additional episodes of fast deterioration after a fever infection or mild head trauma.^1^ Studies show that mutations in the genes that codify the beta sub-unit of the eukaryotic translation initiation factor 2B (eIF2B), a complex that consists of five sub-units, causes the disease in most patients.^2^ A form of leukoencephalopathy that takes place in the prenatal stage and in the first year of life is called Cree’s leukoencephalopathy (CLE), a rare form of brain demyelination which shows homozygote mutation, leading to the replacement of histidine with arginine in the gene eIF2B5.^3^ Findings of the magnetic resonance imaging suggest that, with time, the abnormal white matter disappears, and is replaced by a cerebrospinal fluid.^4^ The physiopathology of the disease is little understood.^5^

Because of the lack of case reports about the disease in Brazil, and after analyzing the scarcity of epidemiological data available, the objective of this study was to describe a case of LVWM in order to improve the knowledge regarding this morbid entity that has a risk of recurrence in future generations.

## CASE DESCRIPTION

Male child, born of a non-consanguineous couple, born at term (38 weeks - gestational age), of a C-section, without intercurrences. The screening for innate errors in metabolism was normal, and included the following conditions: phenylketonuria and other aminoacidopathies, congenital hypothyroidism, sickle cell anemia, and other hemoglobinopathies, congenital adrenal hyperplasia, cystic fibrosis, galactosemia, biotinidase deficiency, congenital toxoplasmosis, Glucose-6-phosphate dehydrogenase deficiency, congenital syphilis, congenital cytomegalovirus, congenital Chagas disease and congenital rubella.

He was discharged from the hospital two days after birth. The infant had history of gastroesophageal reflux since birth, without clinical importance, since he had been gaining weight normally. At the age of 5 months, the infant had conjunctivitis, with sequential fever peaks, and was treated with tobramycin eye drops 0.3%, one drop five times a day. At that time, the infant started to refuse suckling breastfeeding and was very sleepy, progressing to dehydration, dry mouth, increasing body temperature and adipsia. The mother reported having searched for medical care, and the prescription was intravenous sodium chlorine 0.9%, and oxygen therapy, since he presented with low oxygen saturation.

As the days went by, the symptoms became worse, with major increase in sleepiness, so the child was transferred to an intensive care unit (ICU), where he stayed for one week. In that period he underwent cranial magnetic resonance imaging, which identified changes in signal with hyper attenuated T2 predominance, especially affecting the white matter of diffuse and symmetrical aspects. A new magnetic resonance imaging test was conducted after six days, with signs of myelination in the commissural and capsular territories. After the identification of such changes, the diagnosis of LVWM was not established by the physicians, since it required confirmation from genetic tests.

The infant, who had been sleeping for five days, presented with reduced tonus and muscle strength when he woke up, with difficulties of cervical support and hand movements; he was referred to physical therapy follow-up, besides the administration of phenobarbital twice a day, due to the seizures he had while he was in the ICU.

At 11 months, the infant came to the outpatient clinic with a seizure, because the anti-seizure medication had been suspended for less than a week, according to medical orientation. ON that day, his oxygen saturation was 98%, showing tonic-clonic movements, eye sight deviating to the right, and masticatory movements. Oxcarbazepine 300 mg was prescribed for every 12 hours, with hospital discharge.

Thirty days later, the child returned to the emergency room with a seizure, and clozabam was the orientation. Ten days later, the infant returned to the emergency room with a fever, eupneic, and the orientation was to try paracetamol 10 mg every four hours; he was sent home. On the next day, he came back to the emergency room with high and constant fever, for approximately 48 hours, feeling indisposed, prostrated, with irregular appetite, hyperemesis and congested oropharynx, with purulent points in the palatine tonsils. He was hospitalized for the treatment of tonsillitis with antibiotics. The mother reports that, after hospitalization, the patient became worse and no longer responded to treatment, entering a coma state. A culture of the tracheal secretion was carried out, and the isolated micro-organism was *Enterobacter cloacae*. With the days, the patient presented with kidney failure and reduced oxygen saturation, leading to death. The cause of death reported was sepsis due to a systemic inflammatory response, seizing crisis and demyelinating disease. As a result, specific attention was given to the postmortem examination and the genetic test.

The genomic analysis by exome sequencing conducted in deoxyribonucleic acid (DNA), extracted from peripheral blood, was carried out to investigate if the infant presented with genetic variables that could be associated with the regression of the neuropsychomotor development and cavitating leukoencephalopathy. The variant c.896 G>A (ENST0000273783) was identified in homozygosis (two copies) in gene eIF2B5, promoting the replacement of the amino acid arginine, present in the 299 position, with histidine (p.Arg299His). The parents of the infant underwent the same genetic test, which identified the same variant in heterozygosis, thus showing they carried the gene related with LVWM.

## DISCUSSION

LVWM is a hereditary brain disease which affects mainly children. The disease presents itself chronically and progressively, with additional episodes of fast deterioration after fever or mild head trauma.^1^ Studies show that mutations in the gene that codify the beta sub-unit of the eukaryotic translation initiation factor 2B (eIF2B), a complex that consists of five sub-units, causes the disease in most patients.^2^ CLE, a severe variant of LVWM, is caused by a homozygote mutation in the gene eIF2B5.^3^

In 1988, a study conducted in North America with 14 children, with the Cree variant, showed that the affected children showed slight motor delay followed by seizures, hypotonia or spasticity. The proposed cause is a delay in the development or abnormal volume of white matter in the central nervous system. The onset of Cree’s encephalopathy takes place between the ages of 3 and 9 months, and death occurs before the age of 2.^6^ The phenotypical variation of this disease is extremely wide and can affect people of all ages, including patients in the prenatal period, childhood, youth and adulthood.^7^ ^,^ ^8^

The physiopathology of the disease involves deficiency in the maturation of astrocytes, leading the white matter to be more prone to cellular stress. There is no specific treatment, except for the “prevention” of cellular stress. Corticosteroids have sometimes proven to be useful in acute stages. The prognosis seems to be correlated with the age of onset, and the earliest forms are the most severe ones.^9^

Most mutations found in genes eIF2B are “mild”, and leads to the replacement of a single amino acid.^10^ The eIF2B (eukaryotic initiation factor 2B) is a GEF (nucleotide exchange factor) which plays a key role, with the substrate eIF2, in the regulation of the initiation stage of the translation of protein synthesis. The importance of the proper control of eIF2 and eIF2B for a normal physiology is emphasized by the recent involvement of the five genes which codify the five sub-units eIF2B in a severe recessive autosomal neurodegenerative disease, described in young children.^11^ The clinical symptoms include slowly progressive cerebellar ataxia, spasticity, variable optical atrophy, and mental capacities that are relatively preserved. Findings in the magnetic resonance suggest that, with time, there is the progressive disappearance of the abnormal white matter, which is replaced by cerebrospinal fluid.^4^

LVWM is a form of leukoencephalopathy that was first identified in 1993. The authors described three children with fast progressive neurological impact, characterized by ataxia and spasticity, followed by pseudobulbar signs and epilepsy, leading to major motor incapacity in the period of two years.^12^ Afterwards, in 1995, Van Der Knaap et al*.* ^13^ pointed to the probability of the entity to count on a determined genetically recessive inheritance, once, in its series, both genders were represented and, sometimes, more than one sibling was affected. In 1997, Van Der Knaap et al*.* ^14^ published a new series with nine affected individuals, and introduced the denomination used currently, LVWM. The physiopathology of the condition is still little understood^5^.

Studies carried out between 2001 and 2002 identified 5 causal genes and more than 250 patients, and 150 mutations were reported.^1^ ^,^ ^2^ In previous studies with Caucasian patients, the most frequent mutations were in eIF2B5 (containing 57% of all mutations), followed by mutations in genes eIF2B4 and eIF2b2, with 16%. There are also mutations in eIF2B3 and eIF2B1, however, not so often.^14^ ^,^ ^15^ ^,^ ^16^ Based on studies carried out by Van Der Knaap et al*.*,^17^ it was possible to observe a consistent and moderate increase in the glycine amino acid in the cerebrospinal fluid of the five analyzed patients. The increasing content of glycine in the brain is related with the appearance of epileptic crises, however, there are no possible mechanisms established.

There are no detailed studies about the survival rate nor time of life of patients with LVWM. There are reports in which patients diagnosed in early childhood died in the second decade of evolution of the disease.^18^ In a study conducted in China, from December 2013 to December 2015, 49 patients diagnosed with leukoencephalopathy were selected, and the age of onset of symptoms ranged from 20 days to 7 years of age, and the mean age of onset was 1.2 year. The main neurological complaint of these patients included delay/regression in development (27/49 - 55.1%), epilepsy (15/49 - 30.6%), weakness (7/49 - 14.3%), previous clinical ataxia (5/49 - 10.3%), and dystonia (5/49 - 10.3%).^19^ These data show the compatibility of the previous clinic in the infant reported in this case with the diagnosis.

In conclusion, LVWM is a rare condition. The findings in the literature are compatible with the clinical picture of the patient described here. The elucidation of the pathogenesis of the LVWM will be useful to better understand the process of translation of proteins into eukaryotic cells, and provide subsidies for possible therapeutic targets and strategies of treatment in the future.
